# Supplementary material for: Cytoskeletal dynamics regulates stromal invasion behavior of distinct liver cancer subtypes
Source: Commun Biol. 2022 Mar 3;5:202. doi: 10.1038/s42003-022-03121-5 (PMC8894393; doi:10.1038/s42003-022-03121-5)
Supplement: Supplementary file 2 — Supplementary Information [file 42003_2022_3121_MOESM2_ESM.pdf]

Supplementary Information

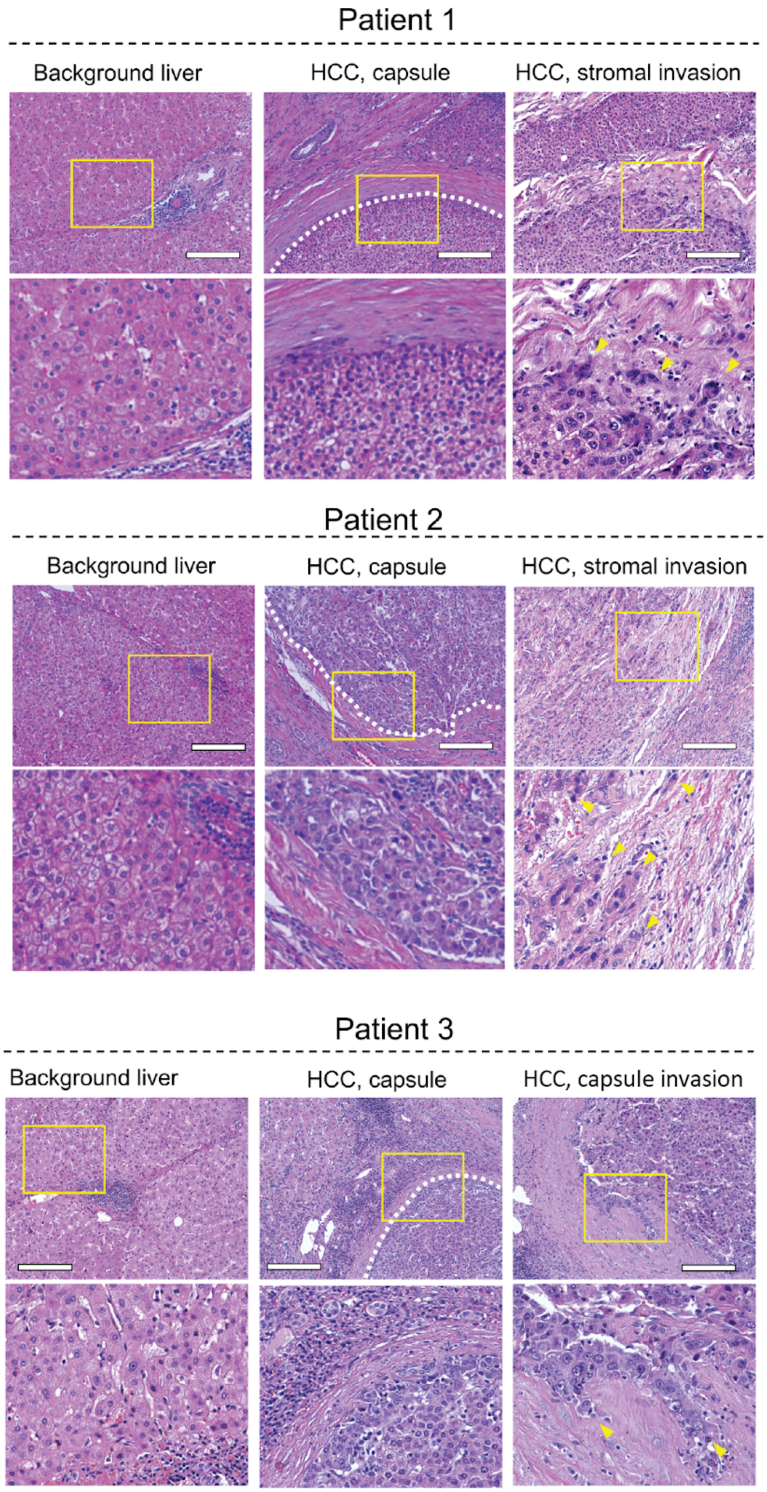

Supplementary Figure 1. Representative HCC histology images of cases displaying multiple invasive phenotypes. Yellow arrows indicate stromally invasive cells. Scale bars: 200 µm.

50

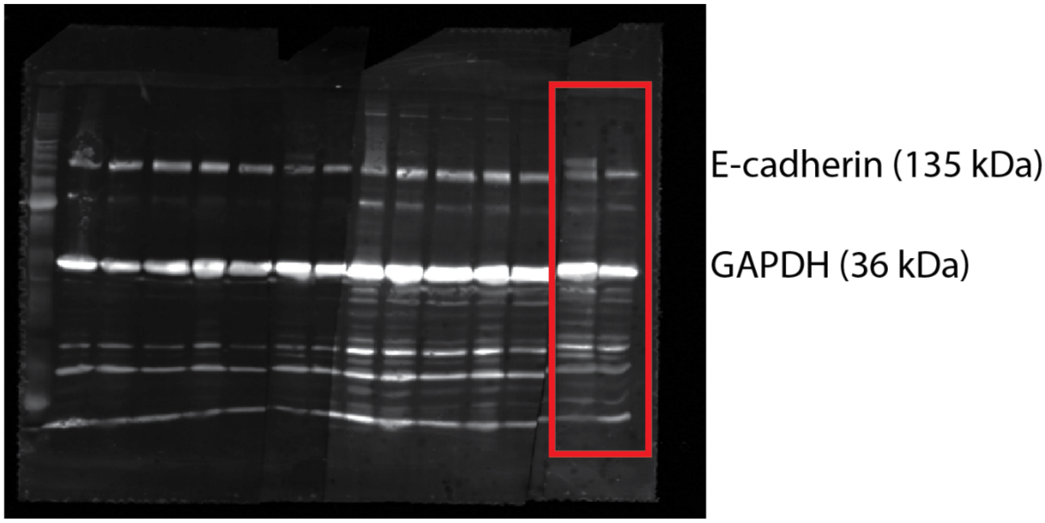

E-cadherin (135 kDa)

GAPDH (36 kDa)

HepG2

SNU-475

50

15

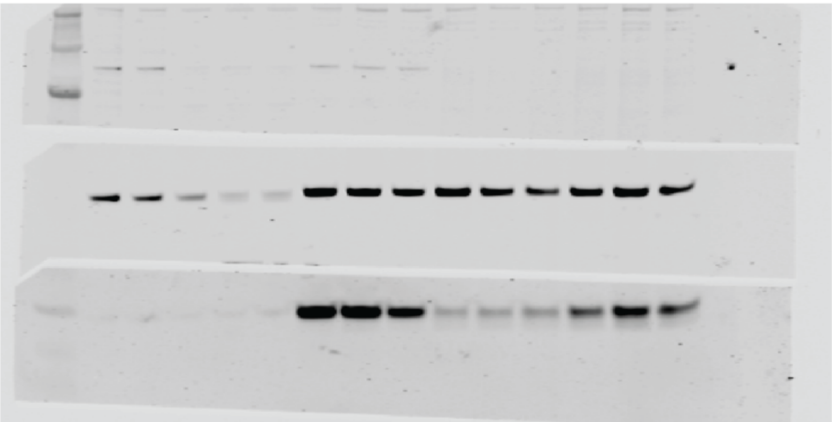

B actin (42 kDa)

pMLC (19 kDa)

HepG2

SNU-475

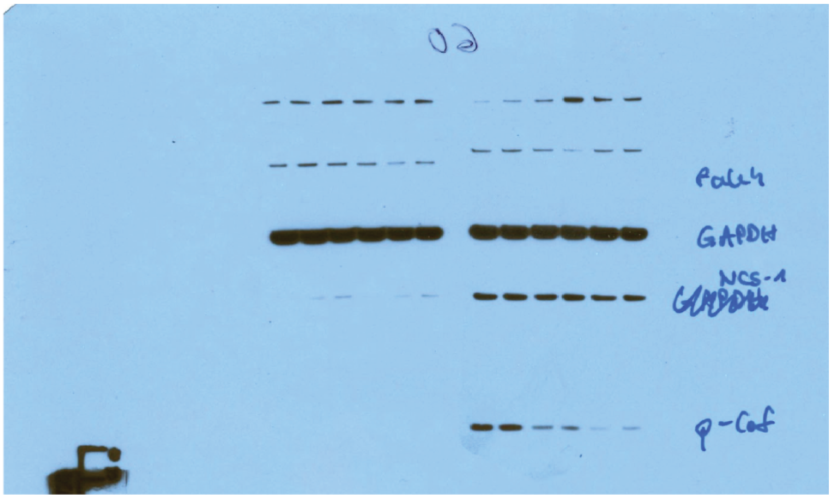

HepG2

SNU-475

Supplementary Figure 2. Western blots SNU-475 and HepG2 cell lines stained for E-cadherin, p-MLC, and p-cof.

HepG2

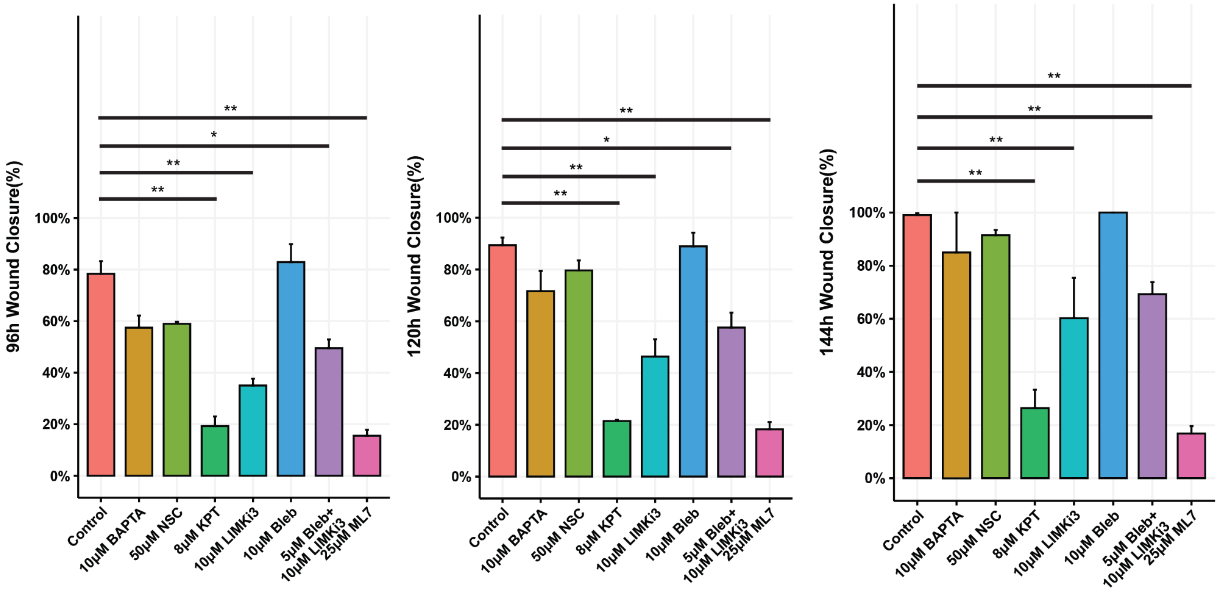

Supplementary Figure 3. Scratch assay analyses. Scratch assay bar plots for HepG2 at 96, 120, 144 hours. Scratch assay bar plots for SNU-475 and HepG2 for all drugs tested. n > 3 independent experiments for all scratch assays performed. One-way ANOVA with Tukey post-hoc testing was performed. Plots show mean ± SEM. *$P < 0.05$, **$P < 0.01$.

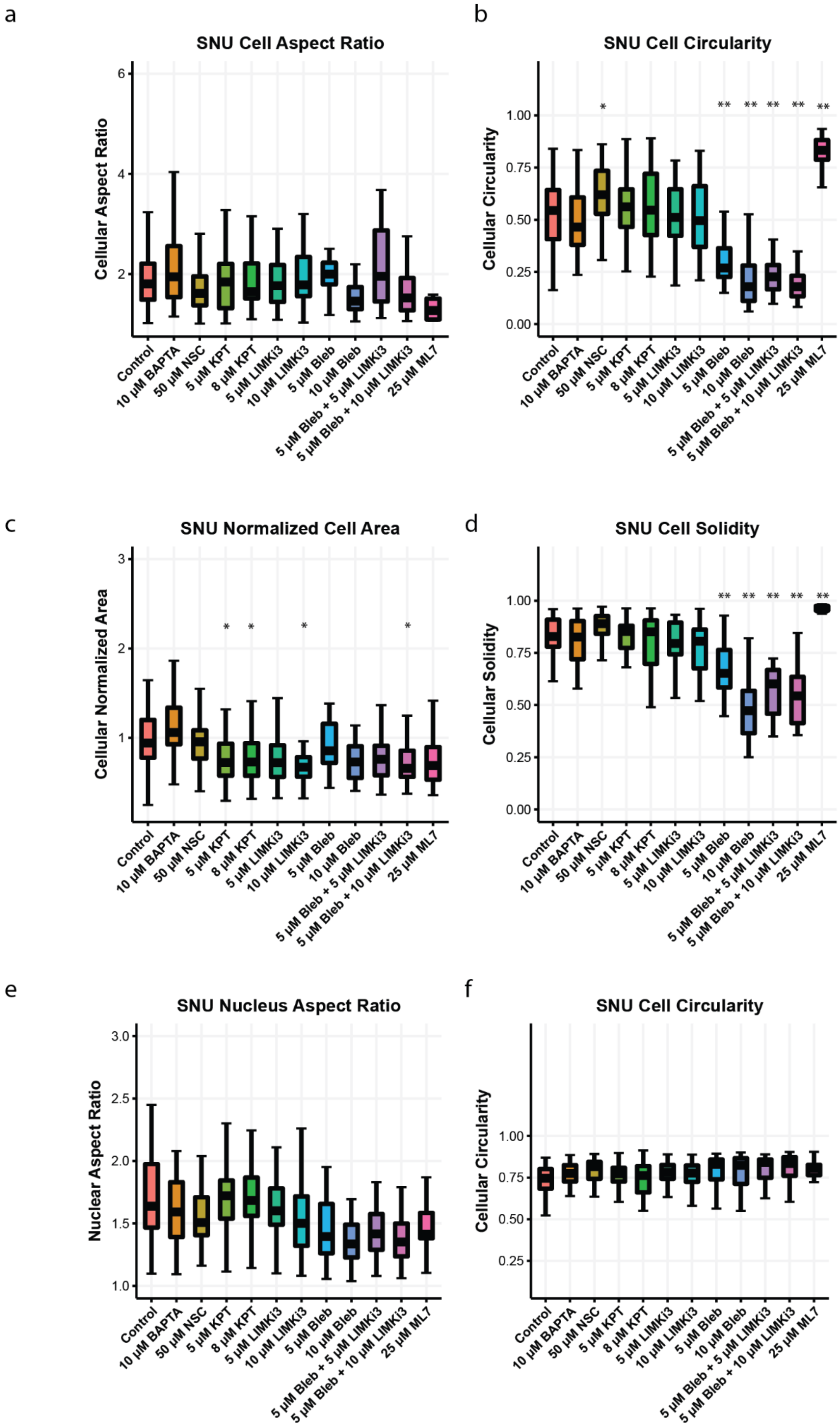
a
SNU Cell Aspect Ratio
Cellular Aspect Ratio
6
4
2
b
SNU Cell Circularity
Cellular Circularity
1.00
0.75
0.50
0.25
0.00
*
**
c
SNU Normalized Cell Area
Cellular Normalized Area
3
2
1
d
SNU Cell Solidity
Cellular Solidity
e
SNU Nucleus Aspect Ratio
Nuclear Aspect Ratio
3.0
2.5
2.0
1.5
1.0
f
SNU Cell Circularity
Cellular Circularity
Control
10 μM BAPTA
50 μM NSC
5 μM KPT
8 μM KPT
5 μM LIMKi3
10 μM LIMKi3
5 μM Bleb
10 μM Bleb
5 μM Bleb + 5 μM LIMKi3
5 μM Bleb + 10 μM LIMKi3
25 μM ML7

Supplementary Figure 4. Cellular (a) aspect ratio, (b) circularity, (c) cell area, and (d) solidity and (e) nuclear aspect ratio and (f) circularity of SNU-475 cells in 2D culture. One-way ANOVA with Tukey post-hoc testing was performed. *$P < 0.05$, **$P < 0.01$.

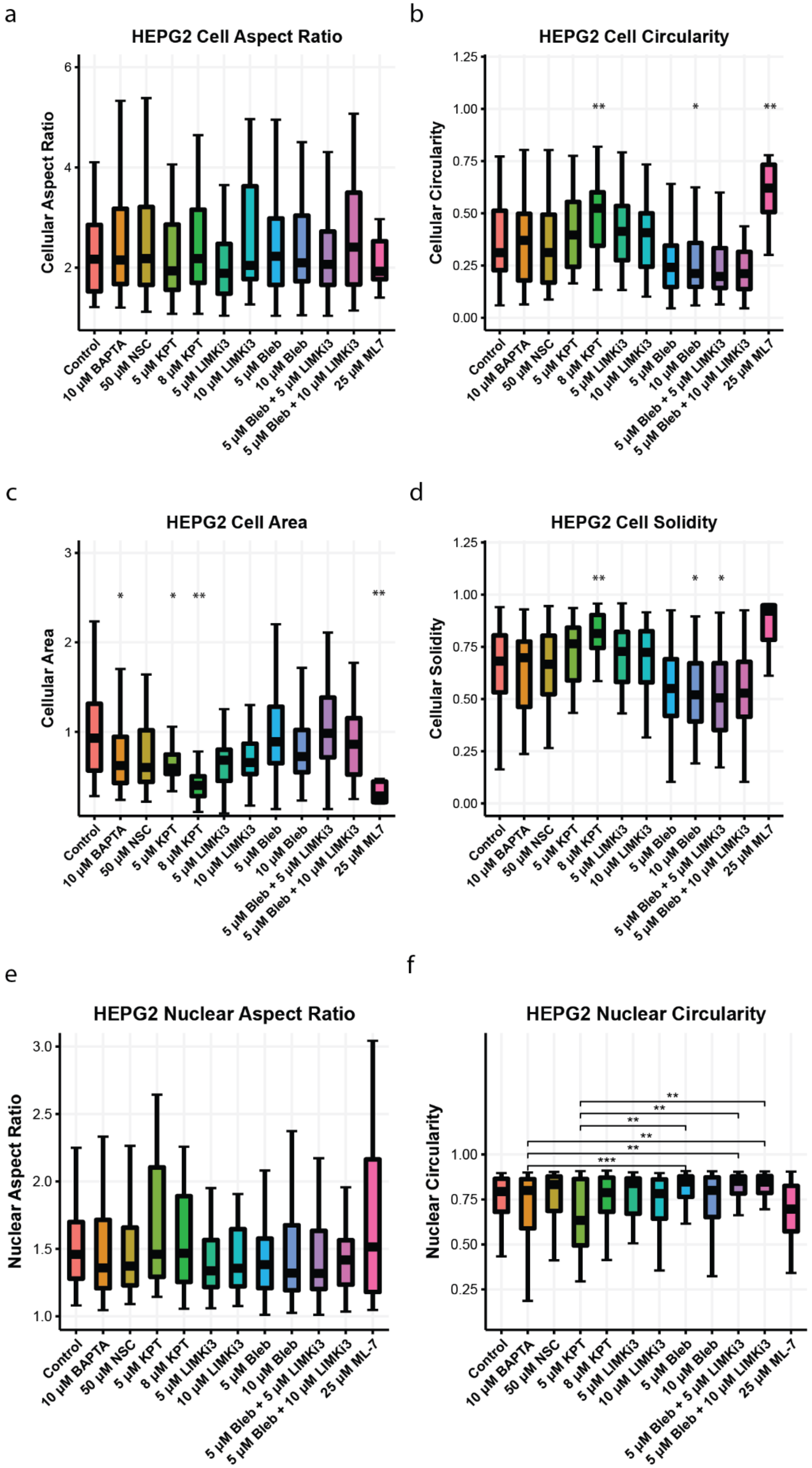
a
HEPG2 Cell Aspect Ratio
Cellular Aspect Ratio
6
4
2
b
HEPG2 Cell Circularity
Cellular Circularity
1.25
1.00
0.75
0.50
0.25
0.00
**
*
**
c
HEPG2 Cell Area
Cellular Area
3
2
1
*
*
**
**
d
HEPG2 Cell Solidity
Cellular Solidity
1.25
1.00
0.75
0.50
0.25
0.00
**
*
*
e
HEPG2 Nuclear Aspect Ratio
Nuclear Aspect Ratio
3.0
2.5
2.0
1.5
1.0
f
HEPG2 Nuclear Circularity
Nuclear Circularity
1.00
0.75
0.50
0.25
**
**
**
**
**
***
Control
10 μM BAPTA
50 μM NSC
5 μM KPT
8 μM KPT
5 μM LIMKi3
10 μM LIMKi3
5 μM Bleb
10 μM Bleb
5 μM Bleb + 5 μM LIMKi3
5 μM Bleb + 10 μM LIMKi3
25 μM ML7
25 μM ML-7

Supplementary Figure 5. Cellular (a) aspect ratio, (b) circularity, (c) cell area, and (d) solidity and (e) nuclear aspect ratio and (f) circularity of HepG2 cells in 2D culture. One-way ANOVA with Tukey post-hoc testing was performed. *$P < 0.05$, **$P < 0.01$

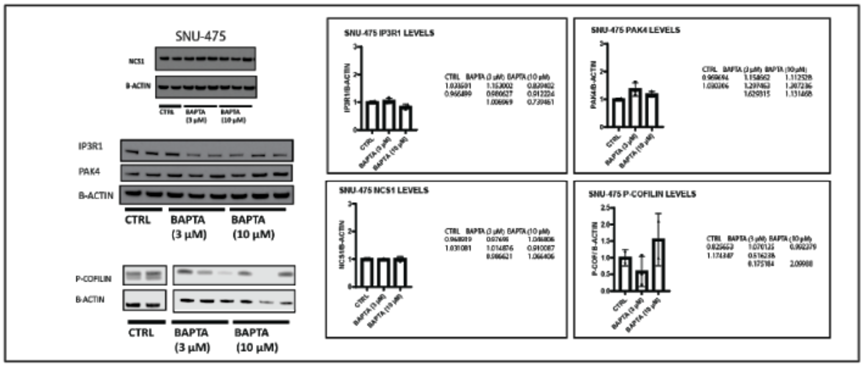
SNU-475
NCS1
B-ACTIN
IP3R1
PAK4
CTRL
BAPTA (3 µM)
BAPTA (10 µM)
P-COFILIN
SNU-475 IP3R1 LEVELS
SNU-475 PAK4 LEVELS
SNU-475 NCS1 LEVELS
SNU-475 P-COFILIN LEVELS

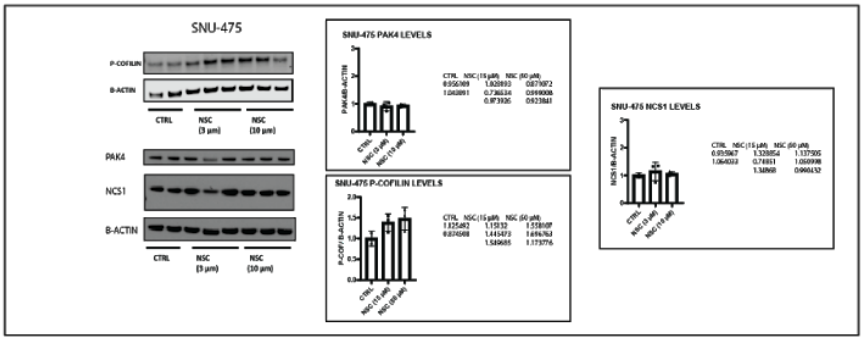
SNU-475
P-COFILIN
B-ACTIN
PAK4
NCS1
CTRL
NSC (3 µm)
NSC (10 µm)
SNU-475 PAK4 LEVELS
SNU-475 P-COFILIN LEVELS
SNU-475 NCS1 LEVELS

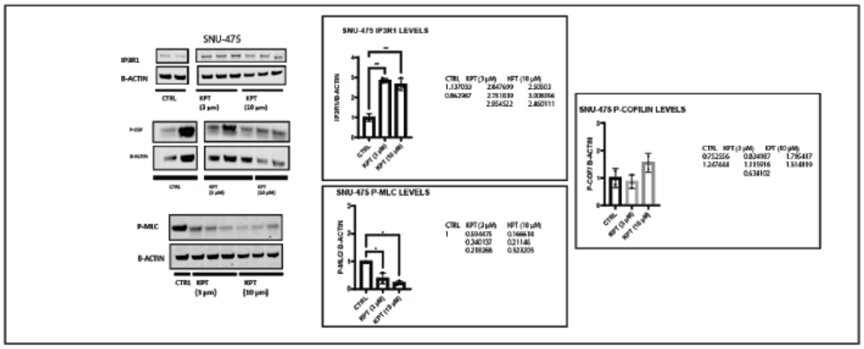
SNU-475
IP3R1
B-ACTIN
P-MLC
CTRL
KPT (3 µm)
KPT (10 µm)
SNU-475 IP3R1 LEVELS
SNU-475 P-MLC LEVELS
SNU-475 P-COFILIN LEVELS

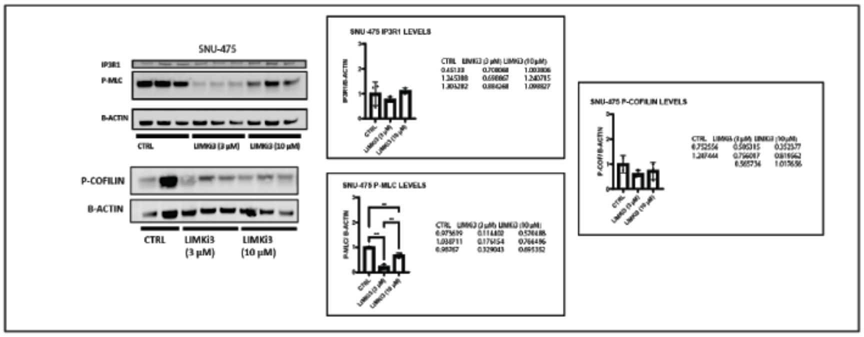
SNU-475
IP3R1
P-MLC
B-ACTIN
P-COFILIN
CTRL
LIMKi3 (3 µM)
LIMKi3 (10 µM)
SNU-475 IP3R1 LEVELS
SNU-475 P-MLC LEVELS
SNU-475 P-COFILIN LEVELS

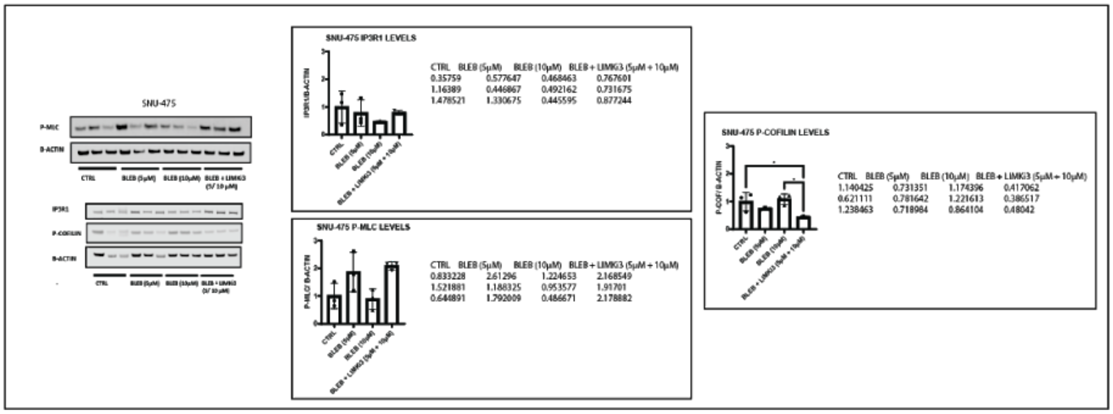
SNU-475
P-MLC
B-ACTIN
IP3R1
P-COFILIN
SNU-475 IP3R1 LEVELS
CTRL BLEB (5µM) BLEB (10µM) BLEB + LIMKi3 (5µM + 10µM)
0.35759 0.577647 0.468463 0.767601
1.16389 0.446867 0.492162 0.731675
1.478521 1.330675 0.445595 0.877244
SNU-475 P-MLC LEVELS
CTRL BLEB (5µM) BLEB (10µM) BLEB + LIMKi3 (5µM + 10µM)
0.833228 2.61296 1.224653 2.168549
1.521881 1.188325 0.953577 1.91701
0.644891 1.792009 0.486671 2.178882
SNU-475 P-COFILIN LEVELS
CTRL BLEB (5µM) BLEB (10µM) BLEB + LIMKi3 (5µM + 10µM)
1.140425 0.731351 1.174396 0.417062
0.621111 0.781642 1.221613 0.386517
1.238463 0.718984 0.864104 0.48042

Supplementary Figure 6. SNU-475 Western Blots after treatment with BAPTA, NSC23766, KPT9274, LIMKi3, Blebbistatin, and Blebbistatin + LIMKi3. Blotting was performed for NCS1, IP3R1, p-cofilin, p-MLC, and B-actin. One-way ANOVA with Tukey post-hoc testing was performed. Plots show mean ± SD.*$P < 0.05$, **$P < 0.01$.

SNU-475 Western Blots

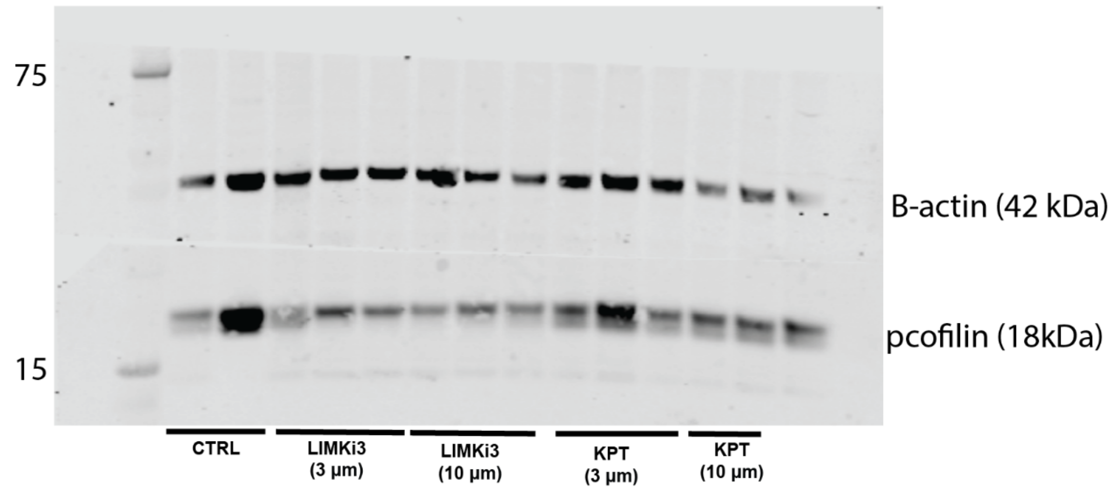

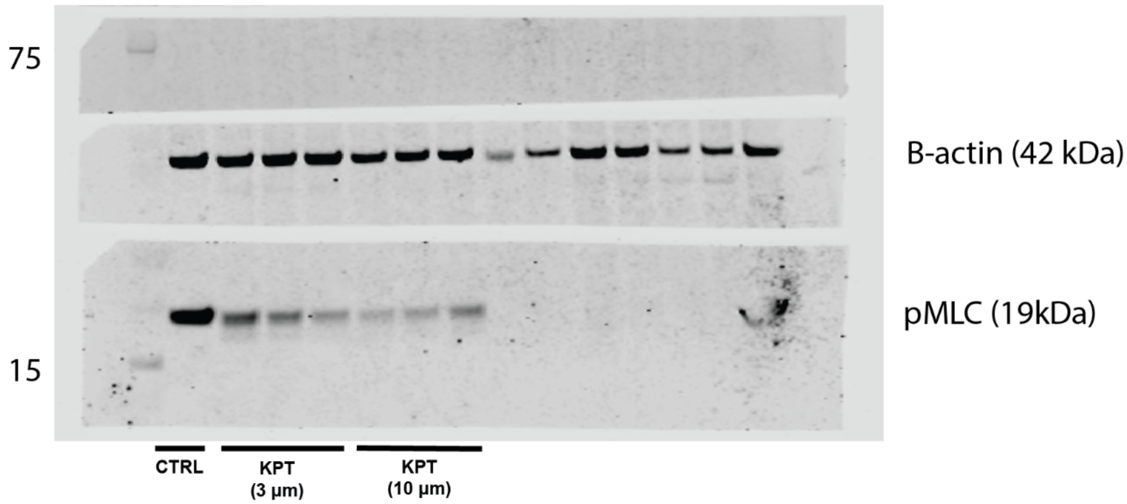

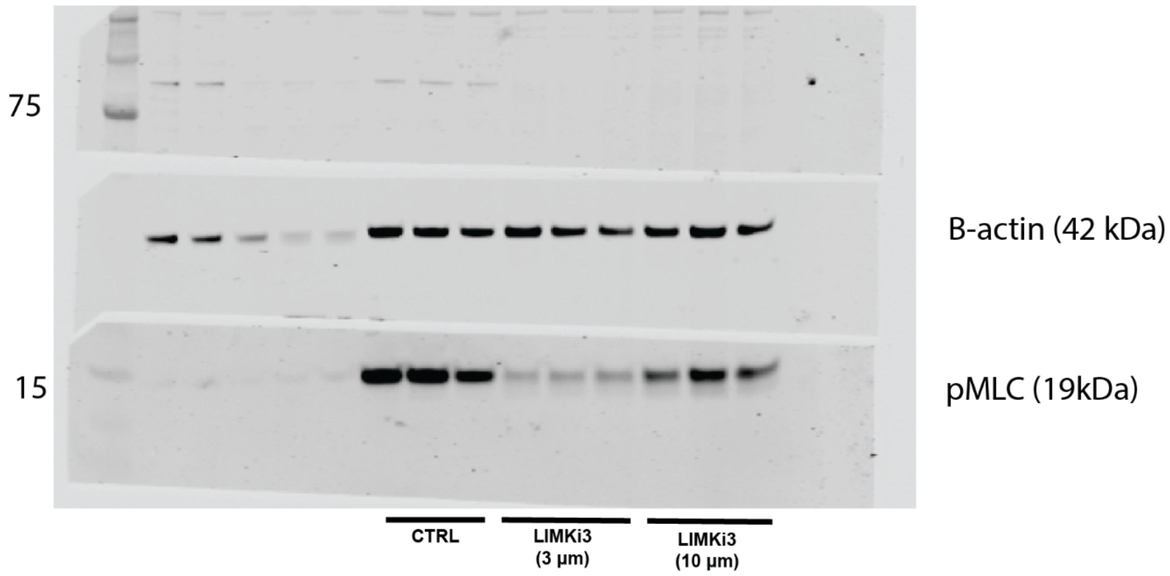

Supplementary Figure 7. Raw Western blots for SNU-475 cells treated with KPT9274 or LIMKi3 stained for p-cofilin and p-MLC.

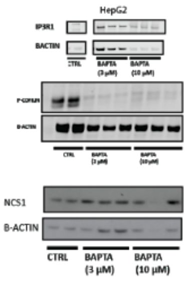

HepG2 IP3R1 LEVELS

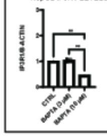

CTRL	BAPTA (3 µM)	BAPTA (10 µM)
1	1.019469	0.460268
	0.960653	0.42587
	1.107109	0.456928

HepG2 P-COFILIN LEVELS

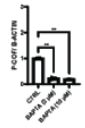

CTRL	BAPTA (3 µM)	BAPTA (10 µM)
0.957891	0.281172	0.204833
1.042109	0.236797	0.222343
	0.156685	0.136364

HepG2 NCS1 LEVELS

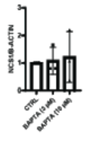

CTRL	BAPTA (3 µM)	BAPTA (10 µM)
0.975076	1.650951	1.069784
1.024924	0.797735	0.375399
	0.793212	2.205652

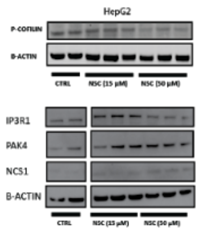

HepG2 IP3R1 LEVELS

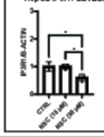

CTRL	NSC (15 µM)	NSC (50 µM)
1.121151	1.045383	0.653259
0.878849	1.063389	0.682511
	0.894815	0.461705

HepG2 PAK4 LEVELS

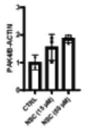

CTRL	NSC (15 µM)	NSC (50 µM)
0.811422	1.010117	1.931732
1.188578	1.888338	1.954033
	1.754652	1.697023

HepG2 P-COFILIN LEVELS

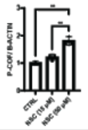

CTRL	NSC (15 µM)	NSC (50 µM)
1.056374	1.082857	1.724656
0.943626	1.268961	1.713477
	1.277447	1.989867

HepG2 NCS1 LEVELS

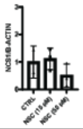

CTRL	NSC (15 µM)	NSC (50 µM)
1.41206	0.687337	0.961972
0.58794	1.457951	0.141385
	1.189028	0.406253

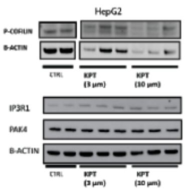

HepG2 IP3R1 LEVELS

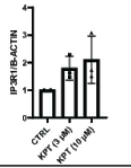

CTRL	KPT (3 µM)	KPT (10 µM)
1.001867	1.471061	3.08073
0.998133	1.621802	1.500465
	2.285963	1.727601

HepG2 PAK4 LEVELS

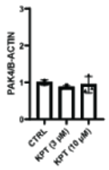

CTRL	KPT (3 µM)	KPT (10 µM)
1.052581	0.843151	1.199592
0.947419	0.922067	0.843867
	0.839154	0.778508

HepG2 P-COFILIN LEVELS

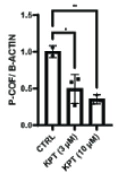

CTRL	KPT (3 µM)	KPT (10 µM)
1.056374	0.268125	0.301062
0.943626	0.627194	0.33514
	0.584051	0.418679

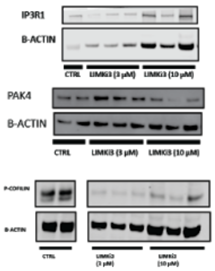

HepG2 IP3R1 LEVELS

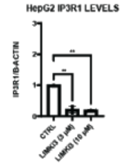

CTRL	LIMKi3 (3 µM)	LIMKi3 (10 µM)
1	0.098357	0.206072
	0.31827	0.157621
	0.21894	0.210834

HepG2 PAK4 LEVELS

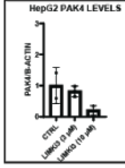

CTRL	LIMKi3 (3 µM)	LIMKi3 (10 µM)
1.411864	0.979756	0.360807
0.588136	0.818755	0.052974
	0.643254	0.194597

HepG2 P-COFILIN LEVELS

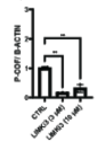

CTRL	LIMKi3 (3 µM)	LIMKi3 (10 µM)
0.957891	0.161208	0.280078
1.042109	0.156598	0.203565
	0.167595	0.465152

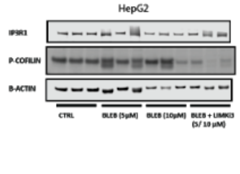

HepG2 IP3R1 LEVELS

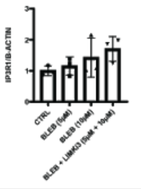

CTRL	BLEB (5µM)	BLEB (10µM)	BLEB + LIMKi3 (5µM + 10µM)
0.953992	1.235643	1.084446	1.248654
1.168788	0.827955	2.154968	1.869223
0.877221	1.384701	1.024727	1.987245

HepG2 P-COFILIN LEVELS

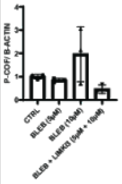

CTRL	BLEB (5µM)	BLEB (10µM)	BLEB + LIMKi3 (5µM + 10µM)
1.069786	0.861939	2.135391	0.673484
1.048355	0.923659	3.044645	0.315978
0.881859	0.759592	0.707815	0.409984

Supplementary Figure 8. HepG2 Western Blots after treatment with BAPTA, NSC23766, KPT9274, LIMKi3, Blebbistatin, and Blebbistatin + LIMKi3. Blotting was performed for NCS1, IP3R1, p-cofilin, p-MLC, and B-actin. One-way ANOVA with Tukey post-hoc testing was performed. Plots show mean ± SD. *$P < 0.05$, **$P < 0.01$.

HepG2 Western Blots

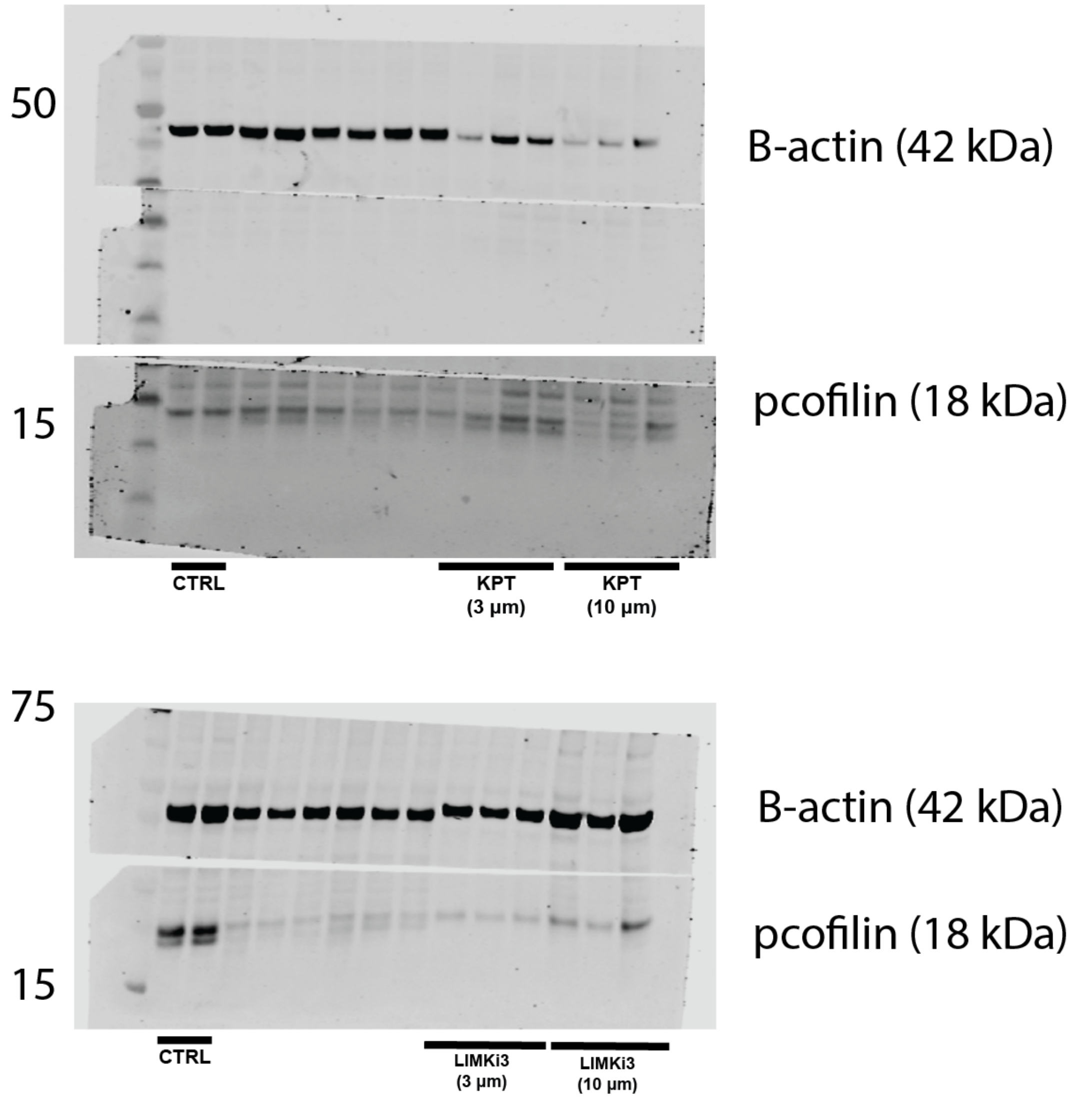

Supplementary Figure 9. Raw Western blots for HepG2 cells treated with KPT9274 or LIMKi3 stained for p-cofilin.

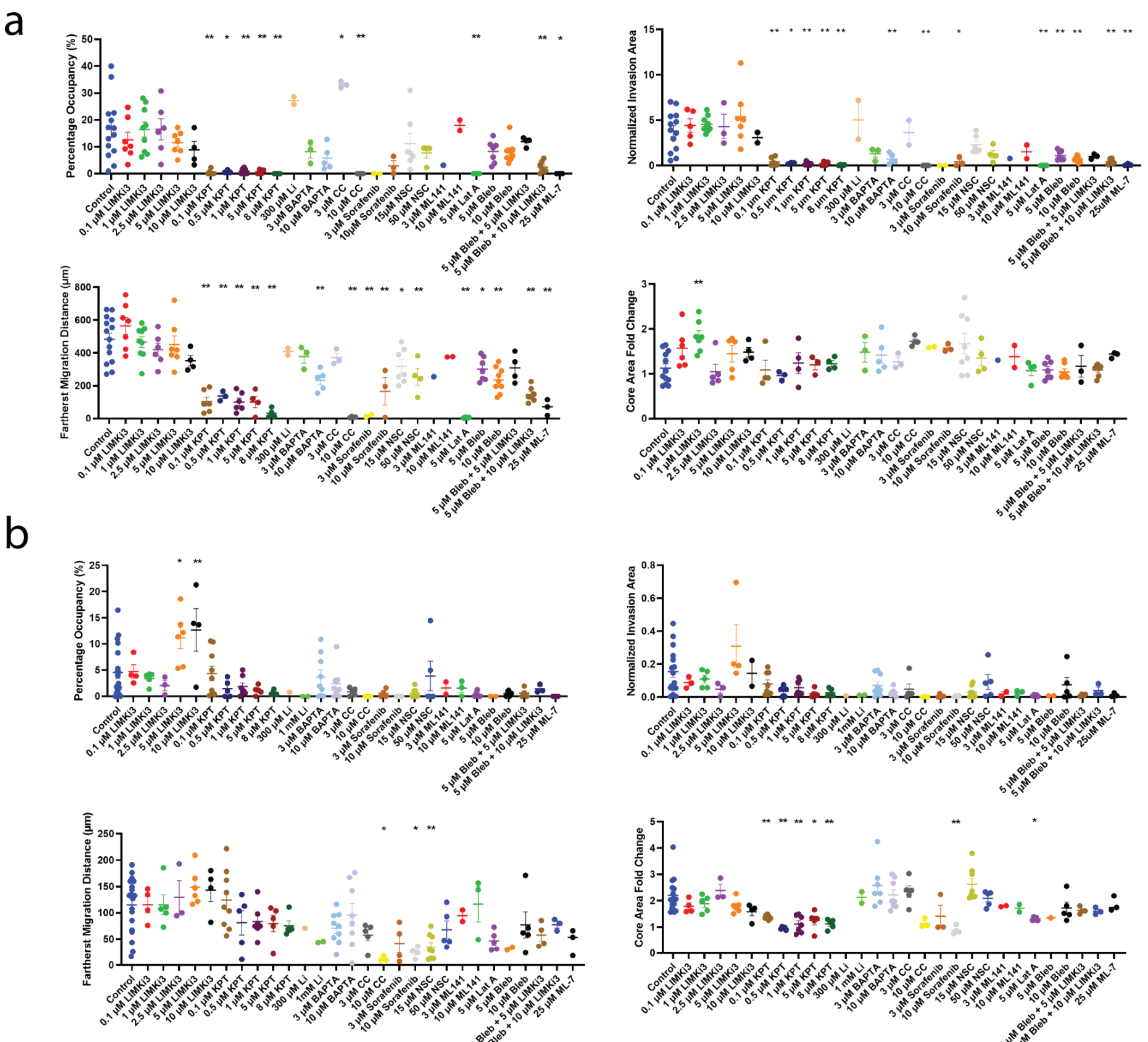

Supplementary Figure 10: 3D Metrics for SNU-475 (A) and HepG2 (B) spheroids 5 days after seeding in collagen gel for all drugs tested. Measurements include percentage occupancy, normalized invasion area, farthest migration distance, and core area fold change. One-way ANOVA with Tukey post-hoc testing was performed. *$P < 0.05$, **$P < 0.01$.

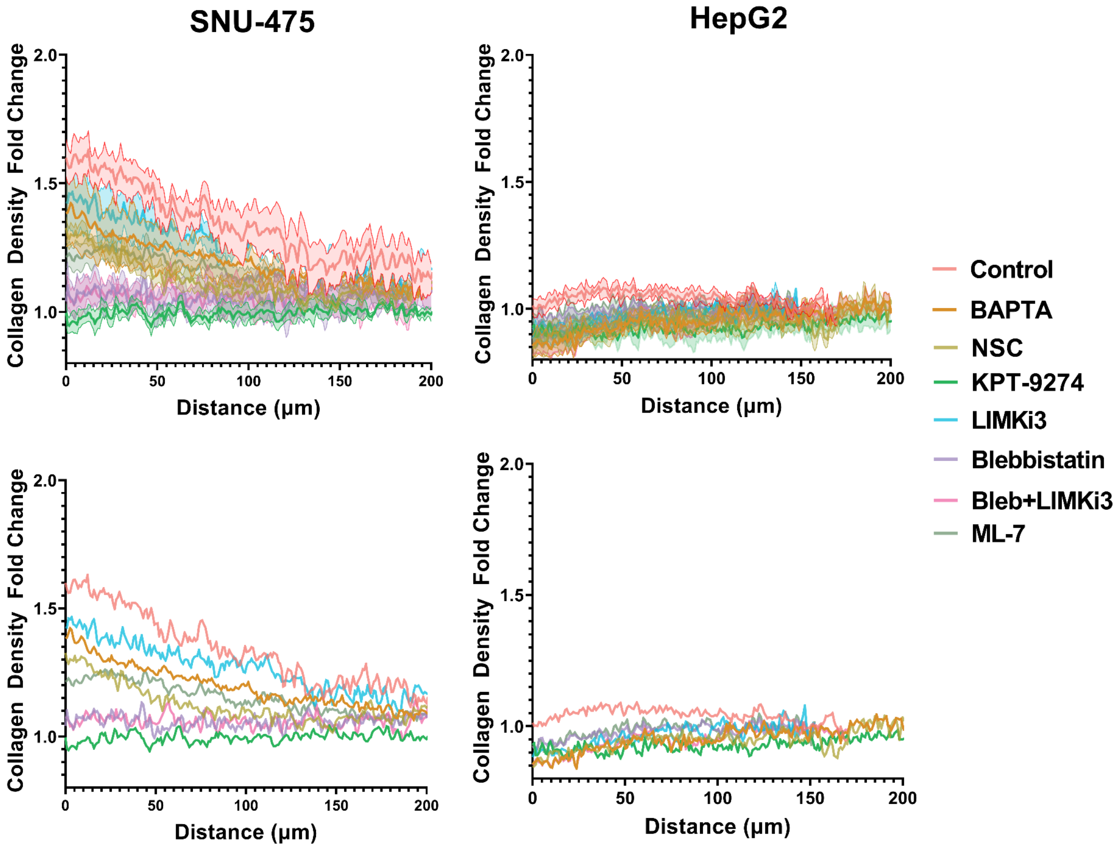

Supplementary Figure 11: Collagen Density profiles for SNU-475 and HepG2 spheroids 5 days after seeding in collagen gel for all drugs tested. Top row shows mean ±SEM while bottom row shows mean. Measurements are normalized to the last 30 data points of each plot profile. The lighter bounds of each curve on the top row are the SEM. n>5 for each condition.

a

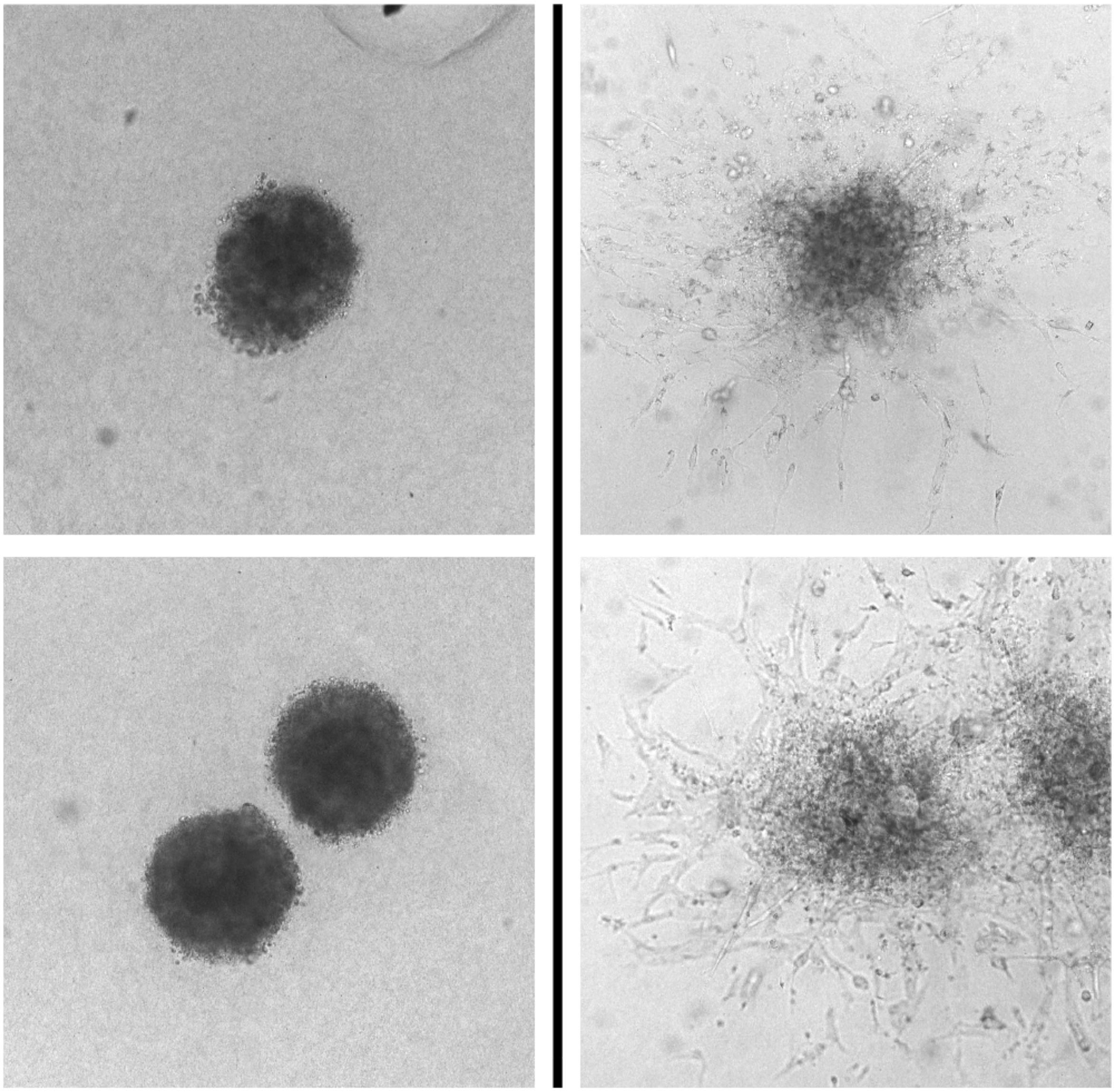

b

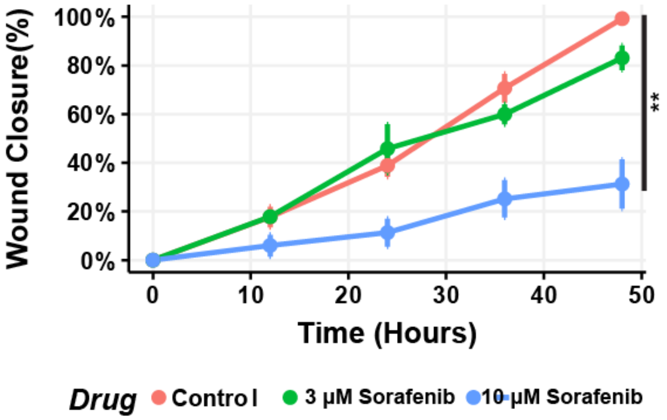
Wound Closure(%)
100%
80%
60%
40%
20%
0%
0
10
20
30
40
50
Time (Hours)
**
Drug
Control
3 μM Sorafenib
10 μM Sorafenib

Supplementary Figure 12. Comparison of SNU-475 spheroids when they are close to bottom and when they are away from the bottom when treated with 3 μM Sorafenib. A) The left two images are away from the bottom, and the right two are close to the rigid bottom. Spheroids are on day 5 of culture. Scale bar: 500 μm. B) 48 hours scratch assay done with sorafenib for SNU-475 cells. 10 μM Sorafenib is able to significantly inhibit 2D wound closure while 3 μM Sorafenib is not. Plots show mean ± SEM.

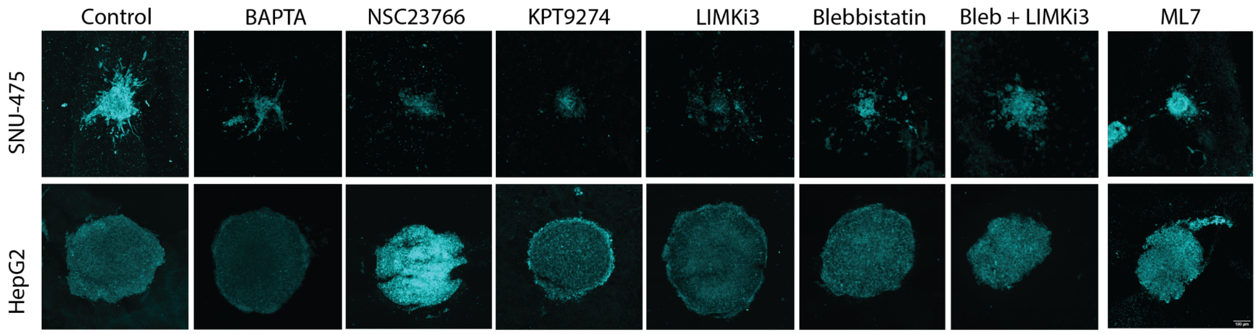

Supplementary Figure 13. Ki67 staining of SNU-475 (top row) and HepG2 (bottom row) spheroids treated with different drugs. Scale bar: 100 μm.

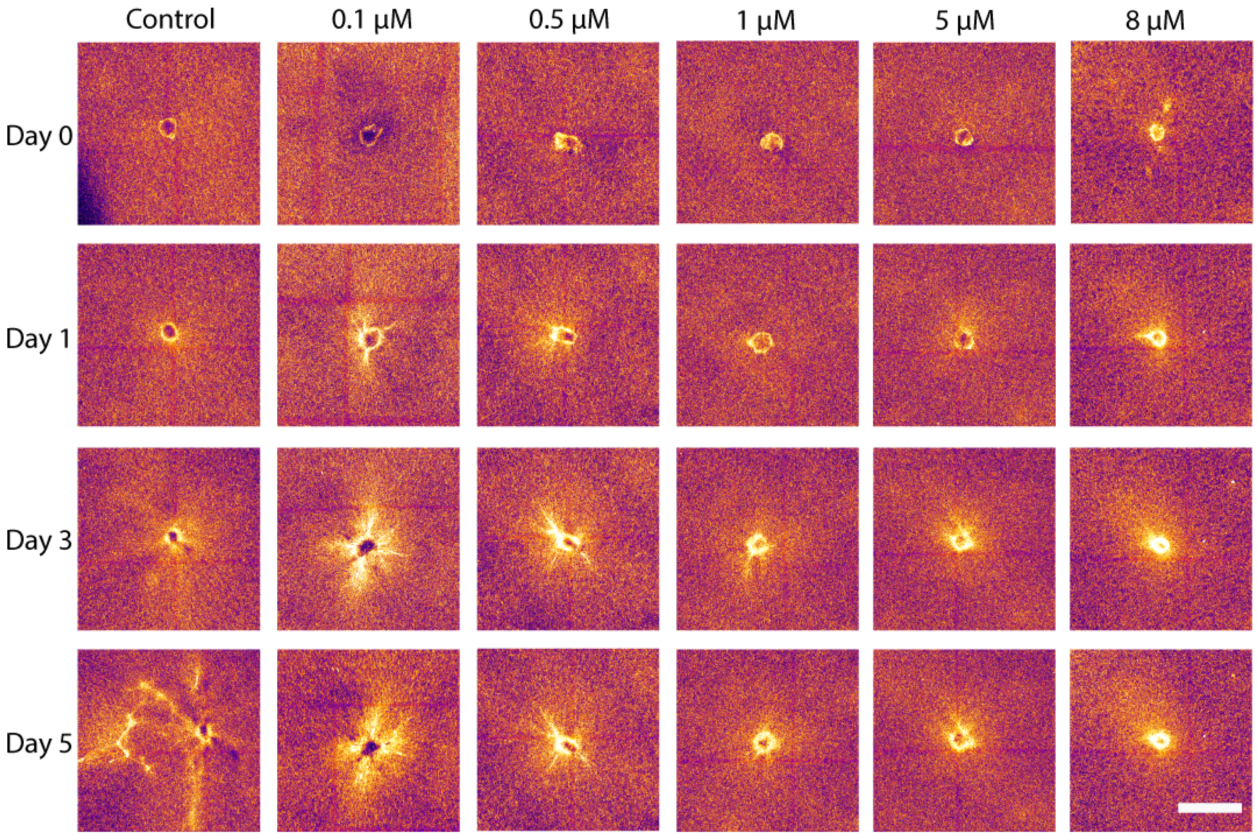

Supplementary Figure 14. Representative images of SNU-475 spheroids treated with different dosages of KPT-9274. Images are acquired using confocal reflectance imaging. Scale bar: 500 μm.

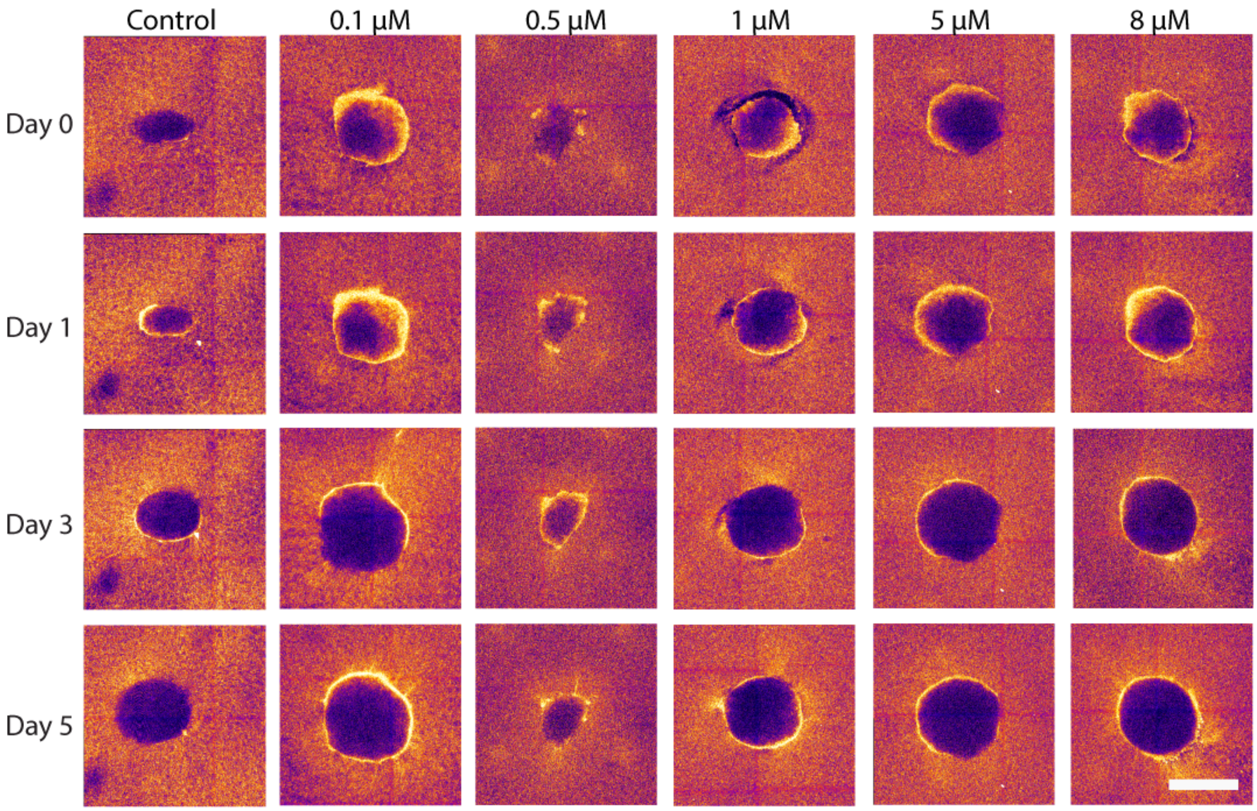

Supplementary Figure 15. Representative images of HepG2 spheroids treated with different dosages of KPT-9274. Images are acquired using confocal reflectance imaging. Scale bar: 500 µm.

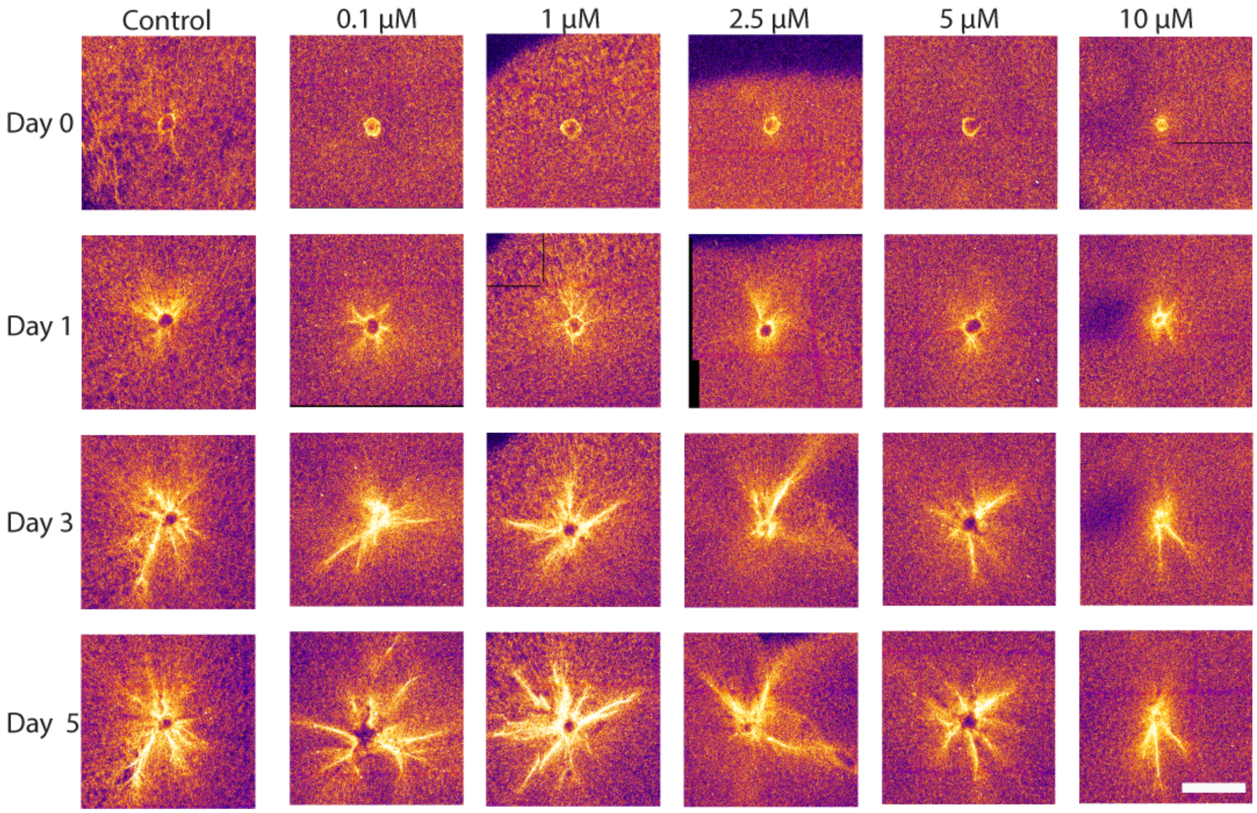

Supplementary Figure 16. Representative images of SNU-475 spheroids treated with different dosages of LIMKi3. Images are acquired using confocal reflectance imaging. Scale bar: 500 µm.

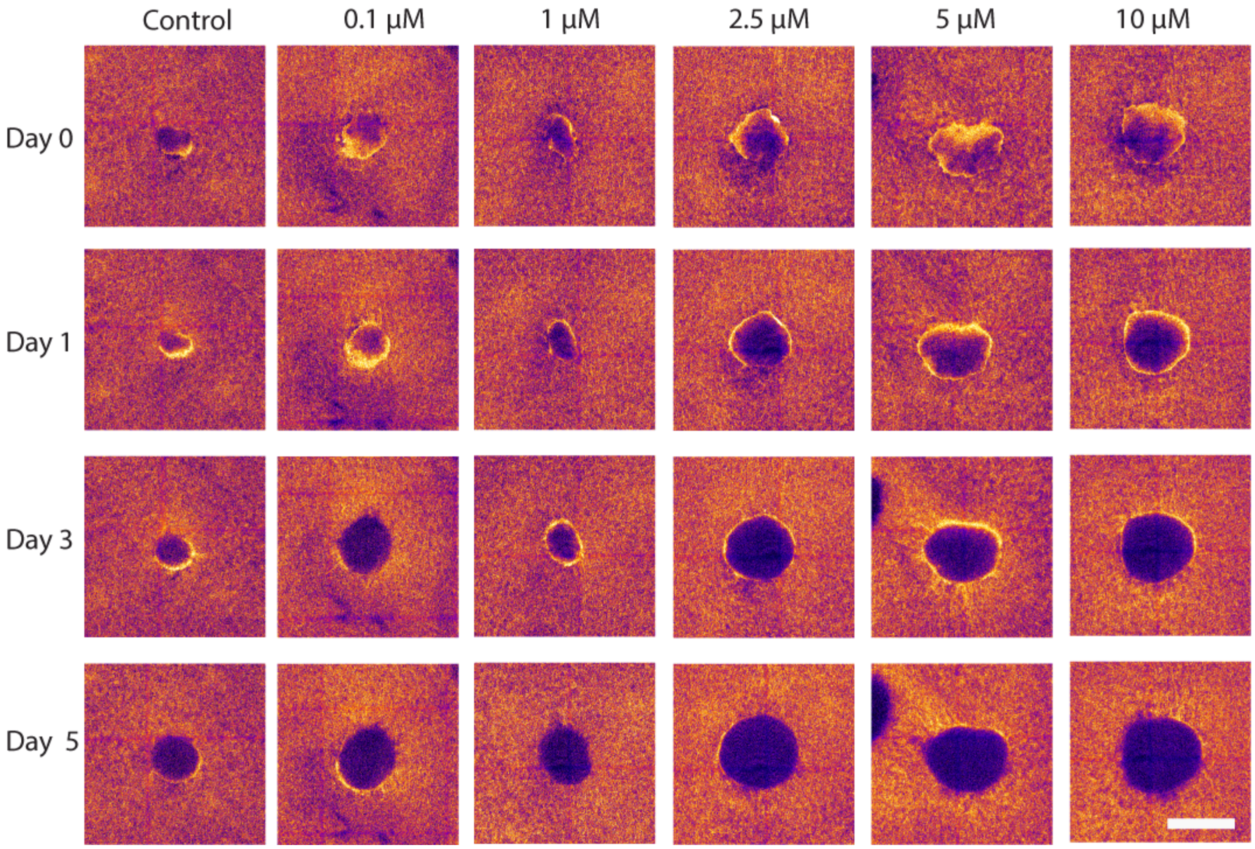

Supplementary Figure 17. Representative images of HepG2 spheroids treated with different dosages of LIMKi3. Images are acquired using confocal reflectance imaging. Scale bar: 500 µm.

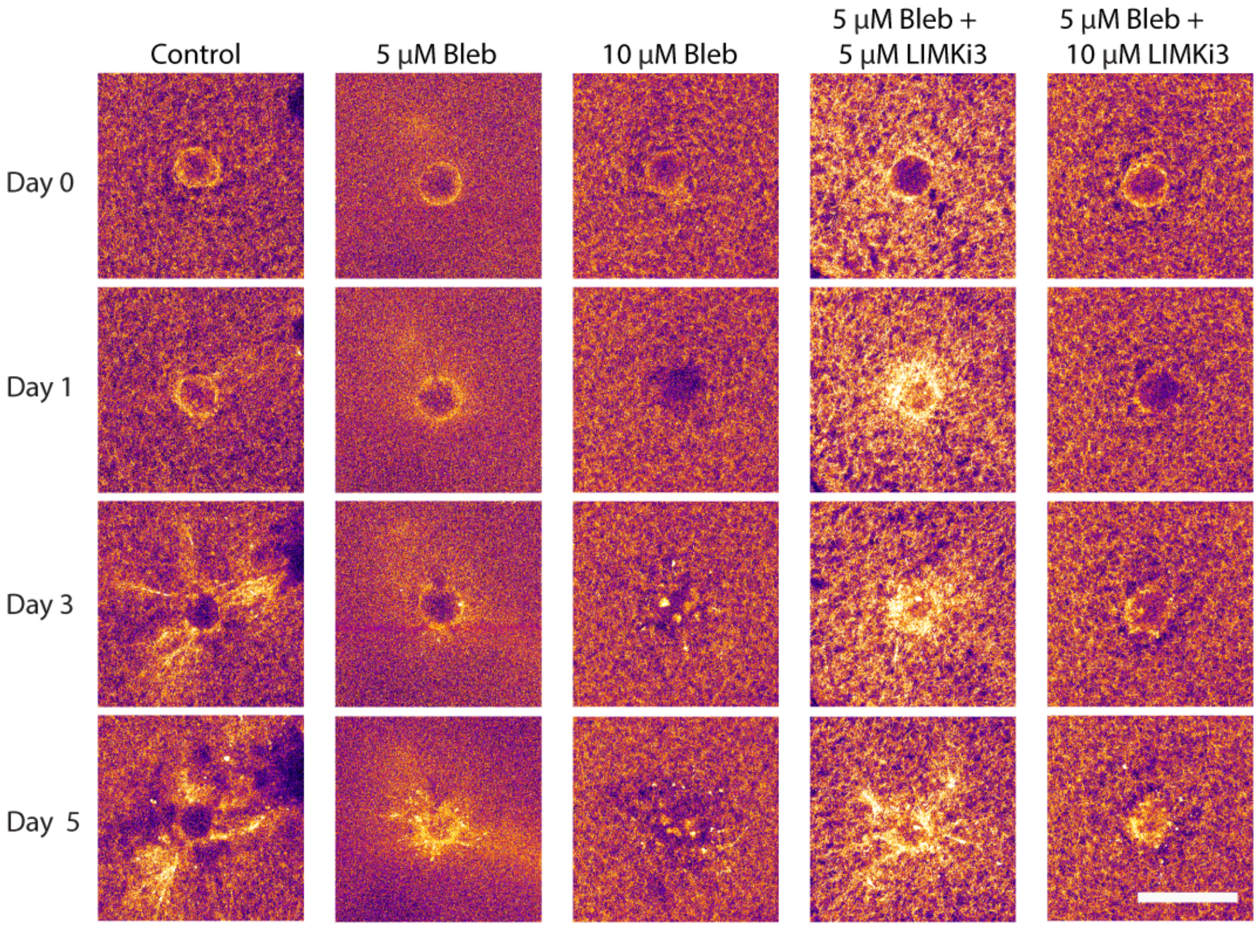

Supplementary Figure 18. Representative images of HepG2 spheroids treated with different dosages and co-treatment of blebbistatin and LIMKi3. Images are acquired using confocal reflectance imaging. Scale bar: 500 μm.

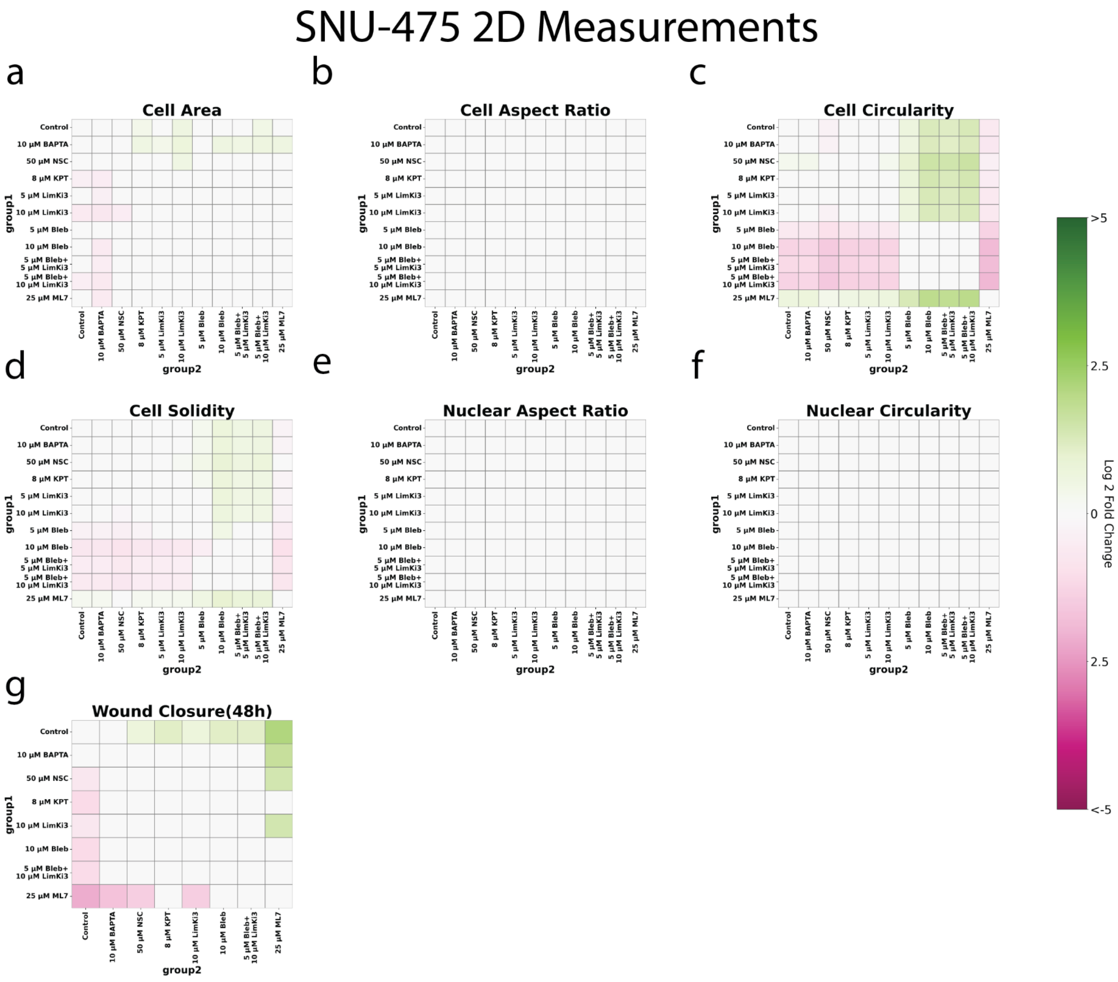

Supplementary Figure 19: 2D Metrics heatmap for SNU-475 cells. Heatmap values are colored if the difference between two conditions is considered significantly different. White indicates no significant difference between conditions. Color intensity is determined by log 2 fold change of Group 1 (y axis) over Group 2 (x axis). Green signifies upregulation while pink signifies down regulation. Measurements include (a) cell area, (b) cell aspect ratio, (c) cell circularity, (d) cell solidity, (e) nuclear aspect ratio, (f) nuclear circularity (g) wound closure after 48 hours. One-way ANOVA with Tukey post-hoc testing was performed.

SNU-475 3D Measurements

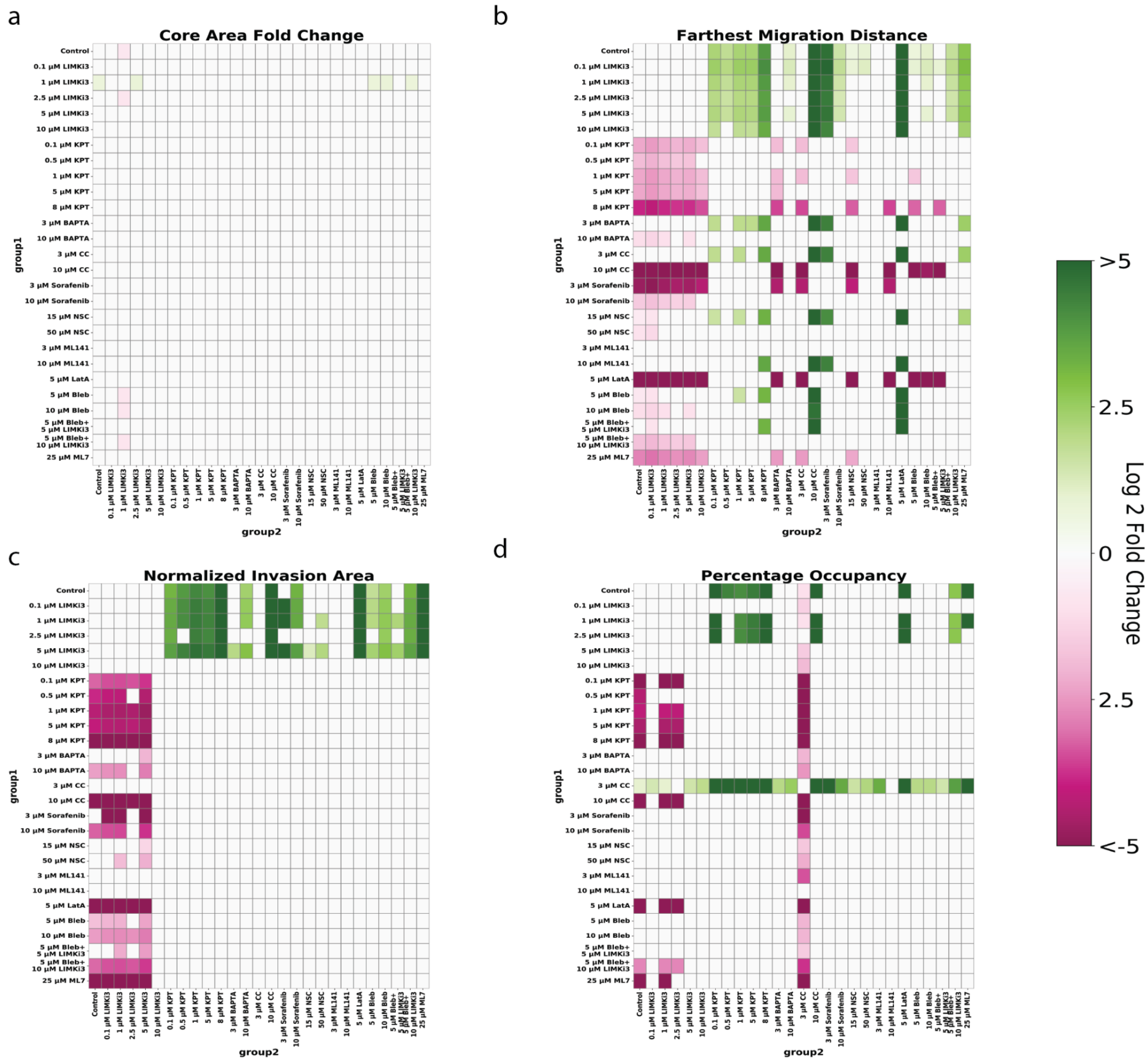

Supplementary Figure 20: 3D Metrics heatmap for SNU-475 spheroids in collagen gels after 5 days. Heatmap values are colored if the difference between two conditions is considered significantly different. White indicates no significant difference between conditions. Color intensity is determined by log 2 fold change of Group 1 (y axis) over Group 2 (x axis). Green signifies upregulation while pink signifies down regulation. Measurements include (a) core area fold change, (b) farthest migration distance, (c) normalized invasion area, and (d) percentage occupancy. One-way ANOVA with Tukey post-hoc testing was performed.

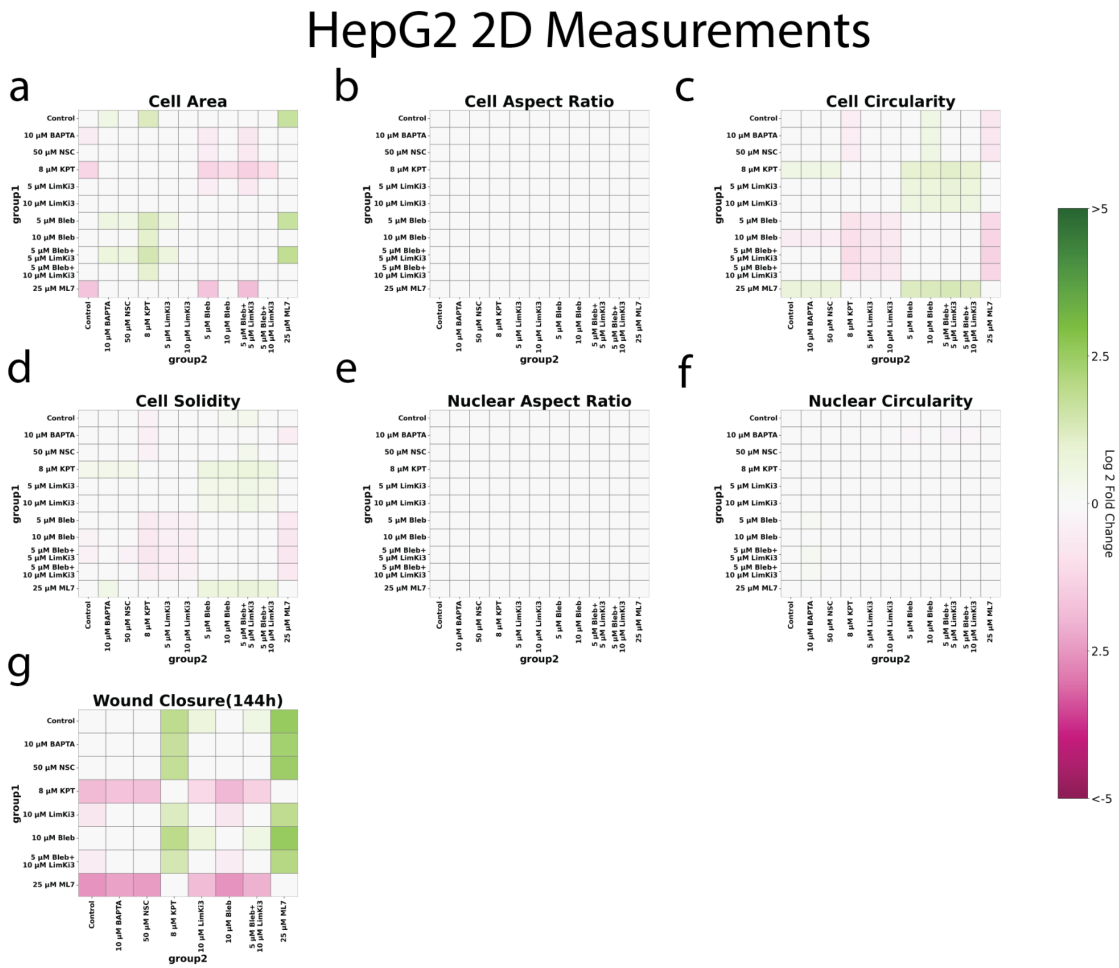

Supplementary Figure 21: 2D Metrics heatmap for HepG2 cells. Heatmap values are colored if the difference between two conditions is considered significantly different. White indicates no significant difference between conditions. Color intensity is determined by log 2 fold change of Group 1 (y axis) over Group 2 (x axis). Green signifies upregulation while pink signifies down regulation. Measurements include (a) cell area, (b) cell aspect ratio, (c) cell circularity, (d) cell solidity, (e) nuclear aspect ratio, (f) nuclear circularity (g) wound closure after 48 hours. One-way ANOVA with Tukey post-hoc testing was performed.

HepG2 3D Measurements

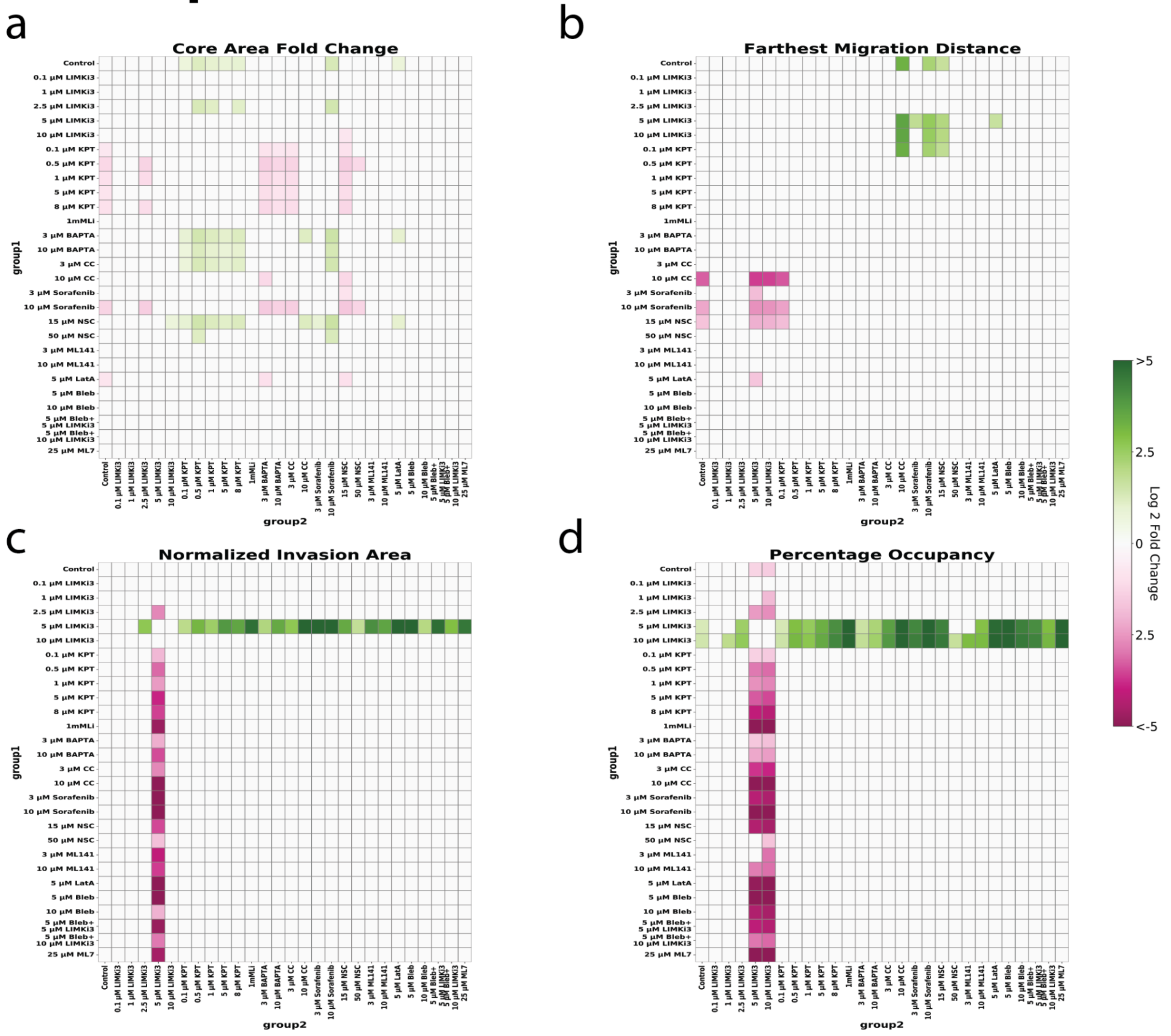

Supplementary Figure 22: 3D Metrics heatmap for HepG2 spheroid in collagen gels after 5 days. Heatmap values are colored if the difference between two conditions is considered significantly different. White indicates no significant difference between conditions. Color intensity is determined by log 2 fold change of Group 1 (y axis) over Group 2 (x axis). Green signifies upregulation while pink signifies down regulation. Measurements include (a) core area fold change, (b) farthest migration distance, (c) normalized invasion area, and (d) percentage occupancy. One-way ANOVA with Tukey post-hoc testing was performed.

a

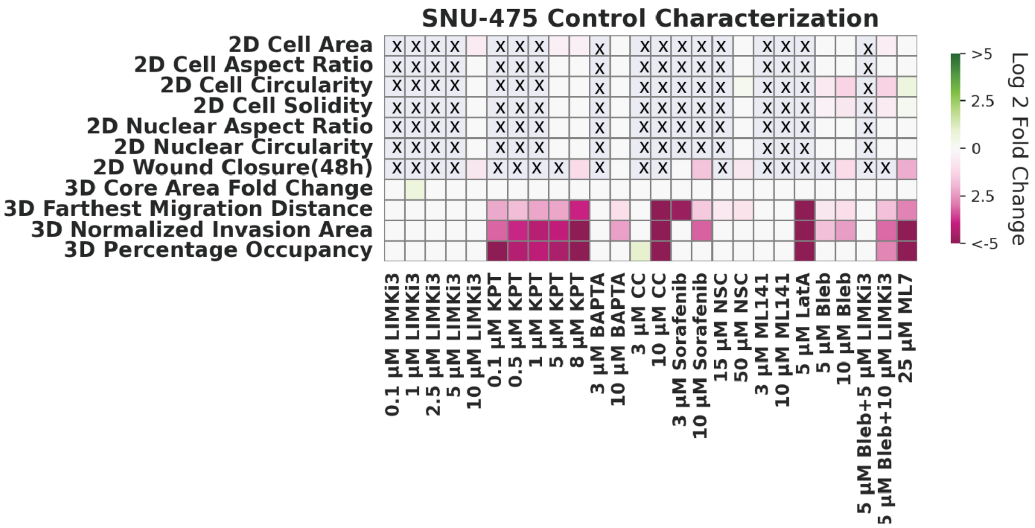

b

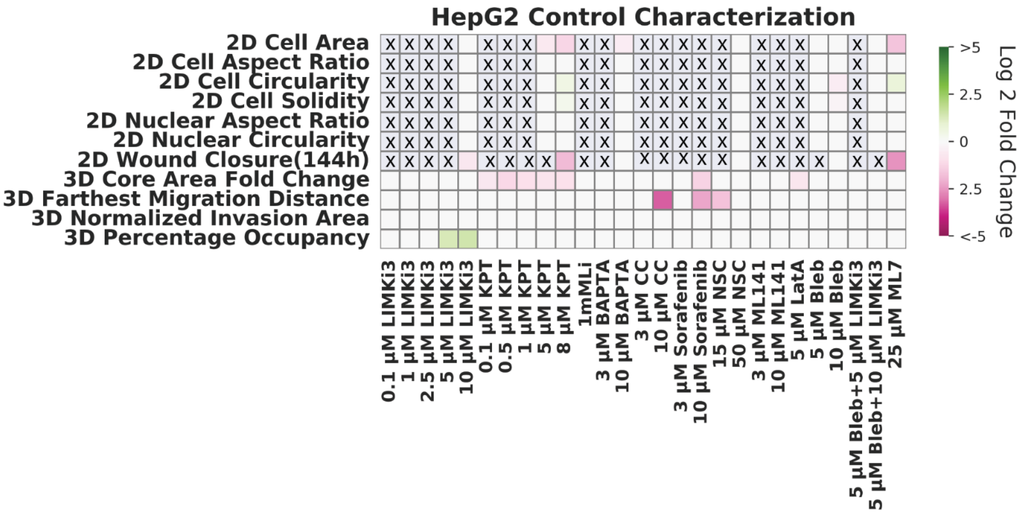

Supplementary Figure 23: Heatmap characterization of all drugs tested for 2D and 3D measurements SNU-475 (a) and HepG2 (b) cell lines relative to control. Heatmap values are colored if the difference between two conditions is considered significantly different. White indicates no significant difference between conditions. Color intensity is determined by log 2 fold change of Group 1 (x axis) over Control. Green signifies upregulation while pink signifies down regulation. X's indicate that the particular metric was not collected for these conditions. One-way ANOVA with Tukey post-hoc testing was performed.

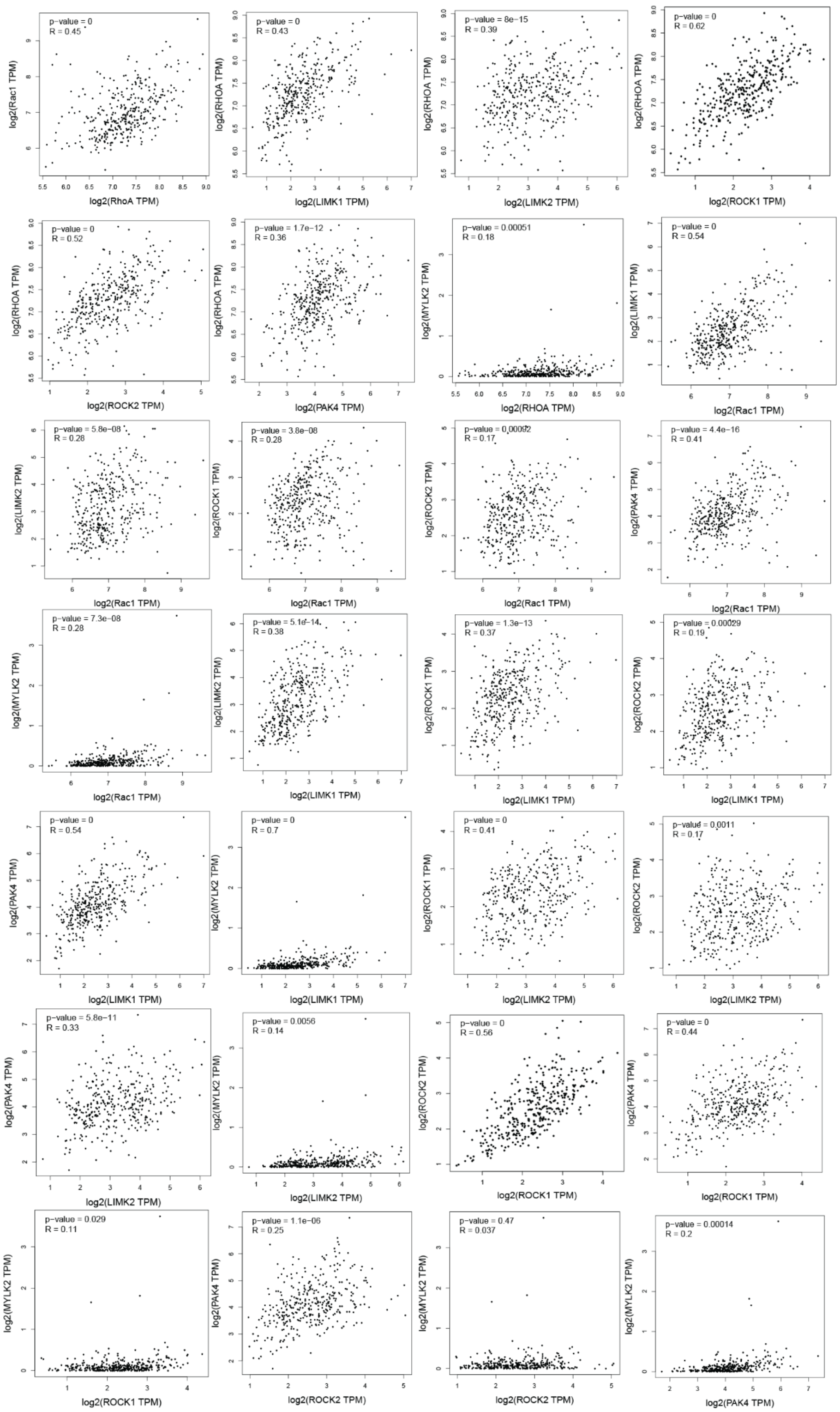

Supplementary Figure 24: Correlation analysis of Rac1, RhoA, LIMK1, LIMK2, ROCK1, ROCK2, and PAK4 in HCC patients from TCGA.

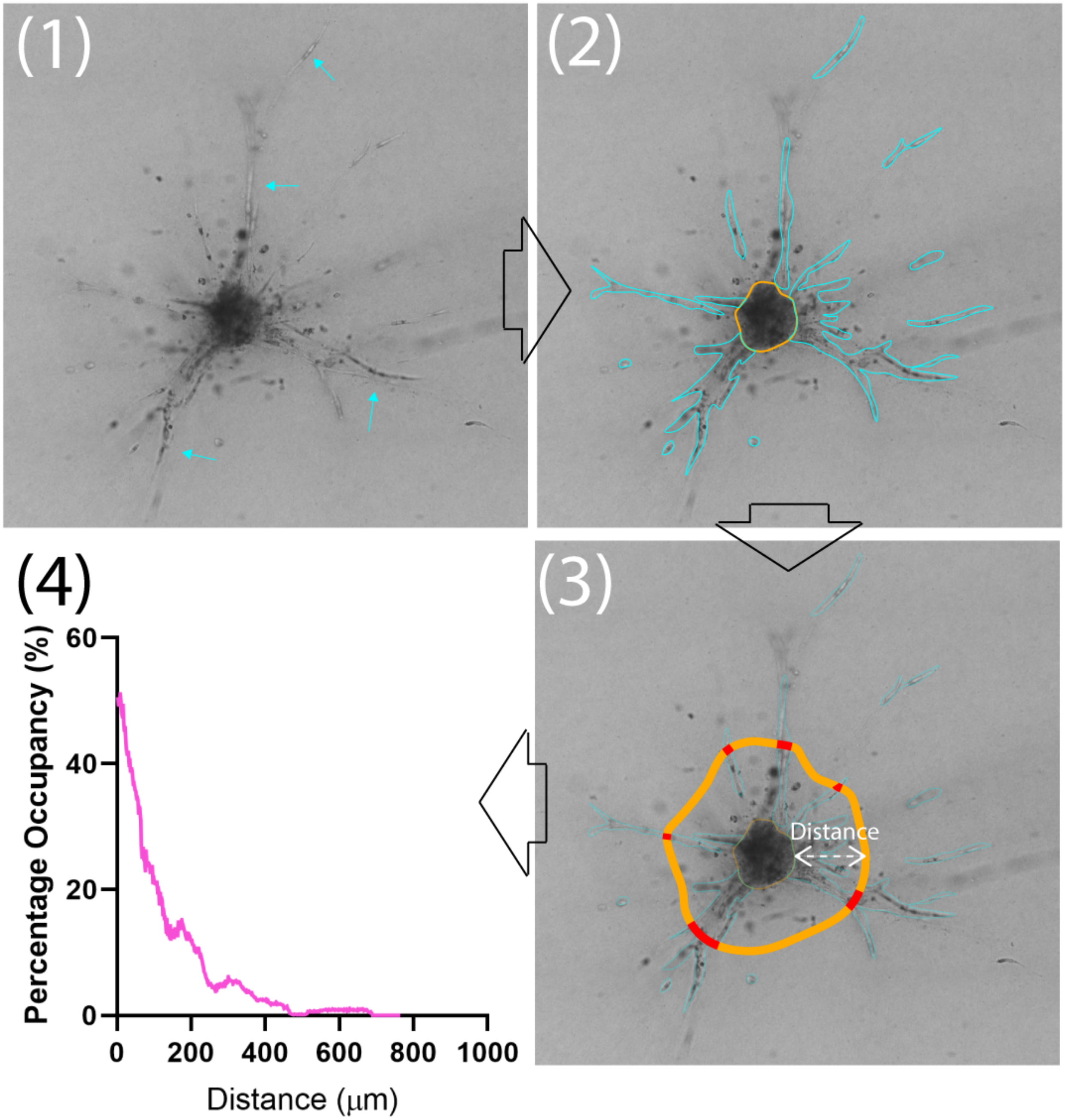

Supplementary Figure 25. Illustration of the analysis of 3D spheroid invasion assay. Steps are the following: (1) In the original images, disseminated cells are identified (teal arrows). (2) Both the disseminated cell regions (teal) and spheroid core are manually traced out. (3) At each distance away from the spheroid periphery, the percentage occupancy (4) is calculated by dividing disseminated regions at that distance (red regions) by the total ring size for that distance (orange ring).
